# Perceived Infant Discomfort Linked to Lower Maternal Oral Health Quality of Life: Results from a Cross-Sectional Study

**DOI:** 10.3390/jcm13195931

**Published:** 2024-10-05

**Authors:** Lisetty Garrido, Inês Rodrigues, Patrícia Lyra, Luís Proença, João Botelho, Sónia Frota, José João Mendes, Vanessa Machado

**Affiliations:** 1 Egas Moniz Interdisciplinary Research Center (CiiEM), Egas Moniz School of Health and Science, 2829-511 Almada, Portugal; lisettymendez@gmail.com (L.G.); ines.rodrigues1042000@gmail.com (I.R.); plyra@egasmoniz.edu.pt (P.L.); lproenca@egasmoniz.edu.pt (L.P.); jbotelho@egasmoniz.edu.pt (J.B.); jmendes@egasmoniz.edu.pt (J.J.M.); 2 Center of Linguistics, School of Arts and Humanities, University of Lisbon, 1600-214 Lisboa, Portugal; sonia.frota@mail.telepac.pt

**Keywords:** infant, digestive discomfort, quality of life, infant colic questionnaire, oral health, postpartum care

## Abstract

**Aim**: We aimed to explore whether there is an association between maternal perceived infant discomfort due to suggestive gastrointestinal alterations and oral-health-related quality of life (OHRQoL) through a survey. **Materials and Methods**: The present study included two main phases involving Portuguese-speaking parents with full-term infants aged 2–12 weeks old who were not previously hospitalized in a neonatal nursery. First, the original French Infant Colic Questionnaire (ColiQ) was translated, cross-culturally adapted and validated to Portuguese (ColiQ-PT). Then, a survey was distributed, and included sociodemographics, the ColiQ-PT, an oral health value scale, OHRQoL, self-perceived periodontal status, and smoking and oral health habits. Data were analyzed through inferential, correlation and multivariate logistic models in this cross-sectional study. **Results**: The ColiQ-PT revealed reliability and validity. From a total of 421 responses, higher infant discomfort was correlated with less maternal professional dental care prioritization (ρ = −0.096, *p* < 0.05). Self-perceived periodontitis correlated with all items of OHRQoL (*p* < 0.001), all seven OHIP-14 domains, and with the physical (*p* < 0.001), psychological (*p* = 0.006), and social (*p* = 0.011) super-domains. While the infant-related score was associated with baby age (*p* = 0.023) and physical pain (*p* = 0.040) related to OHRQoL, the parent score was associated with education (*p* = 0.005), unemployment (*p* = 0.035), and physical pain (*p* = 0.017). The total ColiQ-PT score was significantly associated with more deteriorated social disability related to maternal OHRQoL (ρ = −0.130, *p* < 0.05). **Conclusions**: Perceived infant discomfort seems to be linked to maternal deteriorated OHRQoL. This finding highlights the importance of prioritizing oral health in postpartum care. Further research is needed to explore the mechanisms underlying this association and to develop targeted interventions.

## 1. Introduction

Infant crying (IC), gastrointestinal discomfort, and fussing can have significant impacts on a healthy baby during the early stages of infancy [1], affecting 3% to 40% of newborns worldwide [2,3]. Infant symptoms and the severity of crying are complex to assess, thus tools for measuring and reporting intensity and evolution are currently limited [4,5]. The Infant Colic Questionnaire (ColiQ), a parent self-reported tool, represents a quantitative assessment of the signs parents observe in infants’ behavior associated with crying and its impact on their own quality of life (QoL) [6]. This questionnaire facilitates communication between healthcare professionals and parents [6] in a critical period to the family’s well-being, the postnatal period.

The postnatal period begins with birth and extends up to six weeks postpartum [7]. During this period, mothers, in particular, undergo significant physical, psychological and social changes [8], with impact on their QoL [9,10]. One of the most challenging aspects is the experience of excessive crying of infants [11], which is associated with increased levels of parental stress, depression, anger, and anxiety [11,12], and consequently might have a negative impact on the QoL of both parents and infants [5,13,14]. An observational study that recorded interactions and activities between parents and children, such as feeding, nappy changing, and crying episodes, concluded that the parent-child relationship can be, indeed, negatively affected by excessive crying.

Oral health and its related QoL are vital to the overall well-being [15]. Current approaches define oral health as multidimensional, encompassing physical, psychological, emotional, and social dimensions [16]. Recently, we demonstrated through a conceptual model that oral-health-related quality of life (OHRQoL) plays a significant role as a factor that leads to the use of health services [17]. Any interference, for example, stress-related factors, may result in less professional dental care search, and consequently oral health deterioration. Thus, we hypothesize that the infant crying discomfort, as a stressful agent, may impact parental OHRQoL during the postnatal period, and to the best of our knowledge, this has never been explored.

Hence, the primary aim of this study was to explore whether maternal perceived infant discomfort due to suggestive gastrointestinal alterations can be associated with OHRQoL, via a national survey. We hypothesize that higher levels of perceived infant discomfort would be associated with lower maternal OHRQoL. As a secondary aim, and considering the lack of a Portuguese version of ColiQ, we tested the validity of a translated version (ColiQ-PT).

## 2. Materials and Methods

This study involved two phases: (1) translation of the ColiQ to Portuguese (ColiQ-PT) and testing of the psychometric validity; (2) a survey to collect sociodemographic data, IC status through the ColiQ-PT, the self-perceived oral health values scale (OHVS), OHRQoL, and self-reported measures of periodontitis. This study was conducted in accordance with the Declaration of Helsinki of 1975, revised in 2013, and was approved by the Ethics Committees of the Garcia da Orta Hospital and Egas Moniz School of Health & Science (process number: PT-144/23).

Based on the inclusion criteria of the original ColiQ [6], we included Portuguese-speaking parents who had at least one baby, aged between 2 and 12 weeks. Parents were excluded if their child was preterm or previously hospitalized in the neonatology service [6]. For both phases of the manuscript, the abovementioned inclusion and exclusion criteria were considered.

This study is reported following the Strengthening the Reporting of Observational Studies in Epidemiology (STROBE) guidelines [18].

### 2.1. ColiQ Translation, Reliability and Validation

First, an assessment was made of the applicability of the various questionnaires available in the literature to report the gravity of IC and the impact of excessive crying on parents’ QoL. To this end, the ColiQ, recently developed by Bellaiche et al., was selected because it has proven to be valid and reliable [6].

The ColiQ is a sixteen-question tool, with ten questions concerning the symptom severity (infant score) and six concerning impact severity (parent score). The infant ColiQ subscales dimensions include: ‘‘Quantitative description of crying”, ‘‘Qualitative description of crying”, ‘‘Associated symptoms”, ‘‘Perceived pain related to crying and digestive discomfort”, and ‘‘Infant behavior”. The parent subscales dimensions are as follows: ‘‘Actions and solutions to calm crying”, ‘‘Psychological impact”, ‘‘Impact on life as a couple”, ‘‘Impact on parents’ general state”, ‘‘Impact on daily life”, and ‘‘Overall impact” [6]. Overall, there are three different scores: two subscales (the infant well-being score, the parental well-being score) and the ColiQ total score (the mean of the baby and parental scores) [6].

We contacted the corresponding author of the ColiQ due to restricted intellectual property and submitted a request to Mapi Research Trust (https://eprovide.mapi-trust.org, accessed on 19 February 2024) who authorized the linguistic translation and validation and gave us access to the scaling and scoring of the ColiQ. We recreated the questions in an online Google Forms questionnaire through the original paper for data collection, and then calculated the scores per item per participant.

The interpretation of the ColiQ’s results followed the authors’ instructions: (i) in the infant well-being score, a higher score is linked to a reduced severity of symptoms (infant score); (ii) in the parent well-being score (parent score), a higher score is associated with a lower impact of IC; in the overall ColiQ score, a higher score is related to an overall better state [6].

#### 2.1.1. Sample Size Calculation

The sample size of the validation period was defined according to Terwee et al., ensuring a minimum of five individuals per questionnaire item [19]. The conceptual structure of the ColiQ includes twenty-two items for the Symptom module into ten questions and thirty-four items for the Impact module organized into six questions. The total number of subjects (*n* = 375) was determined by considering the number of parameters and dimensions present in the questionnaire.

#### 2.1.2. Cross-Cultural Translation and Adaptation of ColiQ

The original ColiQ was adapted and translated into Portuguese by two independent translators fluent in Portuguese and English. The translated version was double-checked by the two translators and back-translation into English was performed to confirm the existence of any discrepancy between the original and the translated instrument. A panel of researchers evaluated the questionnaire and any inconsistencies between the translations was discussed (Supplementary Material questionnaire).

**Figure 1:**
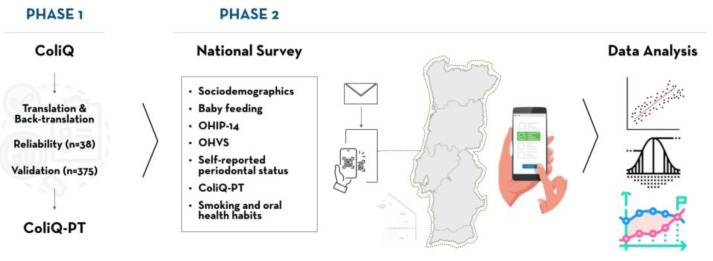
Study organization in two phases. The first phase consisted of translating, adapting and validating the Portuguese version of the ColiQ. The second phase included the national distribution of a survey with QR code reading, with sociodemographics, the ColiQ-PT, an oral health value scale, the oral health impact profile-14 (OHIP-14) to measure oral-health-related quality of life (ORHQoL), self-perceived periodontal status, and smoking and oral health habits questions.

Next, the test–retest phase was conducted for the ColiQ-PT. A random sample of 10% (*n* = 38) of the total sample required for validation who met the inclusion criteria were invited to participate voluntarily and anonymously at the puerperium service of Garcia da Orta Hospital (Figure 1). At this stage, the babies were hours/days old, and parents who agreed to participate were subsequently contacted to complete the online questionnaire. This group of participants did not account for the validation phase. The ColiQ-PT did not require any adjustment based on feedback and the participants were contacted after 24 h to answer the questionnaire again for reliability analysis.

#### 2.1.3. Validation Phase

After the test–retest phase, validity and reliability assessments were conducted between September 2023 and January 2024 on parents’ contacted at the puerperium service of Garcia da Orta Hospital, who accepted to participate and were subsequently contacted to fill in the online questionnaire, and via letter by the Lisbon Baby Lab using data from the National Registration Institute. Written informed consent was obtained from each participant prior to proceeding with the study (Figure 1).

### 2.2. Questionnaires Included in the Survey

This survey was conducted between January and April 2024 and included mothers with full-term babies aged 2 to 12 weeks who had not been previously hospitalized in the neonatology service. A national, anonymous, and representative online survey was administered through the Lisbon Baby Lab, using data from the National Registration Institute for parents of infants in this age range (Figure 1).

For the association between IC and maternal self-reported oral health, a convenient sample method was used. Sociodemographic characteristics and behaviors were collected by self-reported questionnaires. The questionnaire covered questions on the following items: baby date of birth, baby sex, maternal age, educational level (elementary, middle, or higher), marital status (married/union of fact, divorced, single, or widowed), employment status (student, employed, unemployed, or retired), residence municipality, smoking habits (non-smoker, current smoker, or former smoker), oral-hygiene-related behaviors (tooth brushing frequency and interproximal cleaning), and feeding practice for the current baby (breastmilk, mixed feeding, or formula).

Maternal oral health and QoL was assessed using three self-reported questionnaires: (1) the OHVS [20]; (2) the Self-Reported Measures of Periodontitis [21]; and (3) the Oral Health Impact Profile-14 (OHIP-14) [22]. These questionnaires have been shown to be highly sensitive in population-based studies [20,21,22,23].

The OHVS questionnaire measures individuals’ values of oral health and oral-health-related behavior. This instrument consists of 12 items arranged into four subscales that measure relevant OHV dimensions: professional dental care (items 4, 8 and 11); appearance and health (items 3, 7 and 12); flossing (items 2, 5 and 10); and retaining natural teeth (items 1, 6 and 9). Each item is rated using a five-point scale as follows: 1 = “Strongly disagree”, 2 = “Disagree”, 3 = “Neutral”, 4 = “Agree”, and 5 = “Strongly agree”. The total score was calculated by summing the scores for OHVS items with a reverse scoring of items 2, 4, 6, 8, 9, and 11 [20,23].

The Self-Reported Measures of Periodontitis questionnaire includes 13 questions in order to predict cases of periodontitis and severe periodontitis. These questions evaluate several variables such as: 1: Gum disease, 2: Teeth/gum health, 3: Gum treatment, 4: Loose tooth, 5: Lost bone, 6: Tooth appearance, 7: Floss use, 8: Gum bleeding, 9: Gum bleeding in the last 3 months, 10: Loose teeth loss, 11: Gum pain, 12: Gum retraction and 13: Roots visible [21].

The OHIP-14 questionnaire contains seven domains, two questions each: Functional limitation, Physical pain, Psychological discomfort, Physical disability, Psychological disability, Social disability and Disadvantage. The answer types and their scores are as follows: Almost always = 4; Sometimes = 3; Seldom = 2; Rarely = 1; Never = 0. The OHIP-14 scores were determined via the additive method, with higher scores indicating a poorer OHRQoL [22].

### 2.3. Statistical Analyses

#### 2.3.1. ColiQ-PT Reliability and Validity

A reliability analysis of the ColiQ-PT was performed by means of test–retest reliability and internal consistency analysis using 38 participants who completed the ColiQ-PT twice at a 24 h interval. Internal consistency was assessed by calculating Cronbach’s alpha (α) coefficient in the R package version 1.1-1 (R Studio Team 2018) ‘ltm’. An α coefficient of 0.70 was acceptable for the ColiQ-PT items. Test–retest reliability was calculated with the intraclass correlation coefficient (ICC) obtained by the two measurement scores of the participants in the R package ‘irr’ version 0.84.1 (R Studio Team 2018). The ICC values were interpreted as follows: excellent (above 0.9), acceptable (above 0.8), poor (above 0.6), and non-existent (below 0.6). The statistical analysis was performed using the R “plyr” package.

Subsequently, we calculated the overall Kaiser-Meyer-Olkin (KMO) criterion and Bartlett’s test of sphericity to analyze the suitability of the data for factor analysis. Confirmatory factor analysis (CFA) was conducted using the “lavaan” package in RStudio to determine the factorial loads and model fit of each subscale. The maximum likelihood method was employed to compute the model, and differences between the models were examined using chi-square (*χ*^2^) and likelihood ratio tests. To evaluate the fitness of CFA, we used the following criteria: *χ*^2^ /df ratio (considered good with values less than 2), (RMSEA); a good model fit is considered when the value is between 0.05 and 0.10%; 90% confidence interval (CI), confirmatory fit index (CFI) (a CFI value of ≥0.90 indicates a good fit), and goodness-of-fit (GFI) statistics (values of 0.90 or greater indicate well-fitting models) and normed-fit index (NFI) (cut-off criterion of 0.90).

#### 2.3.2. Descriptive and Inferential Analysis of Survey Data

Data were analyzed by using descriptive and inferential statistical procedures. Further, logistic regression analysis was used to model the relationship between the ColiQ-PT values (infant, parent and total scores) and several IC potential risk indicators, based on sociodemographic information and self-reported maternal oral health conditions. A multivariate stepwise approach was conducted. Adjusted Odds-Ratio (OR) and correspondent 95% CI were determined for variables that were included in the final reduced multivariate models. The level of significance was established at 5% in all analyses.

## 3. Results

This section may be divided by subheadings. It should provide a concise and precise description of the experimental results, their interpretation, as well as the experimental conclusions that can be drawn.

### 3.1. Participants’ Description and Reliability of the ColiQ

A total of 38 individuals completed the ColiQ-PT twice, with a 24 h interval. Most of the individuals involved were mothers, with a mean age of 33.1 (±5.9). They had a similar number of girls and boys (20 and 18, respectively) and the babies’ age varied between 14 and 84 days (28.0 ± 18.0).

Overall, the internal consistency was 0.97 (95% CI: 0.91; 0.99), whilst three subscales in infant symptom severity and four subscales in parent impact severity showed excellent coefficient values (Supplementary Table S1). In addition, ICC analyses showed an overall result of 0.93 (95% CI: [0.86–0.96]; *p* < 0.0001), with both parental and infant scores reporting excellent reliability values above 0.9. Nominally, three subscales had excellent reliability (Psychological impact, Impact on the life of a couple, Impact on daily life) and five subscales had acceptable reliability (signs Qualitative description of crying, Perceived pain related to crying and digestive discomfort, Infant behavior, Impact on the general condition of the parent, Global impact) (Supplementary Table S1).

### 3.2. Participants’ Description and Construct Validity 

The validation phase involved 375 participants recruited between September 2023 and January 2024, and they were predominantly female (96.5% vs. 3.5%) of 33.2 (±5.2) years old. There were similar numbers of girls and boys (194 and 181, respectively). At the time of questionnaire response, the babies’ age varied between 14 and 84 days (mean 56.3 ± 22.9).

The overall Kaiser-Meyer-Olkin criterion value was 0.907, ergo these data are probably suitable for factor analysis. The Bartlett’s test of sphericity was *χ*^2^ (45) = 1401.98, *p* < 0.001, considered significant at an alpha level of 5%, thus the data are suitable for factor analysis.

When analyzing the results of ColiQ-PT, sign 4 “Perceived pain related to crying and digestive discomfort” had the highest average score of 48.67 (±29.67), while impact 4 “Impact on the life of a couple” had the lowest score with 24.59 (±30.72) (Supplementary Table S2).

#### 3.2.1. Factor Validity

The CFA analysis attested the unifactorial structure of the ColiQ-PT (Supplementary Table S2). The first-order unifactorial model resulted in an adequate model fit: GFI = 0.947; CFI = 0.946; RMSEA = 0.076; 90% CI [0.060–0.093] (Supplementary Table S2).

#### 3.2.2. Relationships between ColiQ-PT Components

We assessed the correlation between the items of the ColiQ-PT through Spearman’s rank correlation coefficient. There were 11 out of 45 significant correlations (24.4% of all correlations) (Supplementary Table S3).

### 3.3. Survey Results

All 421 participants were recruited between January and April 2024, and only mothers were included in the survey. The participants had a mean age of 32.9 (±4.1), predominantly with higher education (88.1%), married (84.8%), and non-smokers (75.3%). Almost 80% reported brushing their teeth twice or more per day, although only 29% of the subjects performed interproximal cleaning in all teeth. Additionally, the participants consisted of 52.5% boys and 47.5% girls, with a mean age of 48.3 days (±21.8), and the majority of the samples reported a breastfeeding practice (75.3%) (Table 1).

The ColiQ-PT results did not show any significant association with infant sex, type of infant feeding practice, maternal OHRQoL affectance and self-reported periodontal status (*p* > 0.05) (Table 2). Yet, self-perceived periodontitis showed significant higher OHRQoL deterioration in the total OHIP-14 score (*p* < 0.001) and all seven OHIP-14 domains (Table 3), and in the three super-domains, physical (*p* < 0.001), psychological (*p* = 0.006) and social (*p* = 0.011). No type of infant feeding practice significantly impacted on OHRQoL (Table 3).

When analyzing the correlation levels of the ColiQ-PT with the OHVS and the OHIP-14 (Table 4), higher infant discomfort correlated with less maternal professional dental care prioritization as measured through the ‘Professional Dental Care’ of the OHVS (ρ = −0.096, *p* < 0.05). The parent item was also significantly correlated with deteriorated ‘physical pain’ (ρ = −0.101 *p* < 0.05), ‘psychological disability’ (ρ = −0.107 *p* < 0.05), ‘social disability’ (ρ = −0.130 *p* < 0.05) and the ‘Physical superdomain’ (ρ = −0.101 *p* < 0.05). The infant score correlated with deteriorated ‘social disability’ (ρ = −0.106 *p* < 0.05). Similarly, the total ColiQ-PT score significantly correlated with more deteriorated social disability related to maternal ORHQoL ‘social disability’ (ρ = −0.130 *p* < 0.05).

The multivariate logistic regression analysis (Table 5) identified several significant predictors of IC, defined by lower ColiQ-PT values and dichotomized as high/low based on median scores for infant, parent, and total categories. Among infant-related factors, the age of the baby in days was found to be significantly associated with colic, with an (OR) of 0.987 (95% CI: 0.977–0.999, *p* = 0.023), indicating that younger infants had a slightly higher risk of colic. Additionally, the physical pain domain score from the OHIP-14 showed a positive association with the infant well-being score (OR = 1.149, 95% CI: 1.006–1.311, *p* = 0.040), suggesting that higher physical pain increased the likelihood of severe symptoms of IC.

For the parent-related score, higher levels of education were significantly associated with increased odds of infant colic (OR = 4.474, 95% CI: 1.555–12.870, *p* = 0.005), as was being unemployed (OR = 4.897, 95% CI: 1.114–21.515, *p* = 0.035). Moreover, parental physical pain, as measured by the OHIP-14, also showed a significant positive association with colic (OR = 1.098, 95% CI: 1.017–1.185, *p* = 0.017).

In the total score, the social disability domain of the OHIP-14 was significantly associated with infant colic (OR = 1.515, 95% CI: 1.039–2.208, *p* = 0.031), indicating that greater social disability was linked to higher odds of colic. These findings highlight the multifactorial nature of infant colic, implicating both infant-specific and parental factors in its development.

## 4. Discussion

In this national survey, we were able to study the link between infant discomfort and maternal OHRQoL following the successful translation and validation of the ColiQ-PT. Our results show that perceived infant discomfort is associated with diminished maternal OHRQoL, particularly in the social domains of the OHIP-14, and with self-reported periodontal state as a major confounding factor in this link.

The findings emphasize the extensive impact of infant well-being on maternal health and quality of life, indicating that stress related to infant discomfort can affect oral health perceptions and experiences beyond general health. Moreover, mothers with poorer self-reported periodontal health may be more prone to experiencing reduced oral-health-related quality of life when faced with infant discomfort, as demonstrated by significantly higher deterioration in the total OHIP-14 score, its seven domains, and the three super-domains: physical, psychological, and social.

Periodontitis has an estimated prevalence of 40% in pregnant women [24], is consistently linked to adverse pregnancy outcomes [25] and its impact may extend postpartum and beyond. These insights call for a holistic approach in healthcare that considers both infant and maternal health, emphasizing the need for supportive measures that address both physical and psychosocial aspects to improve overall family well-being. This could potentially lead to better health outcomes for both mothers and their infants, by recognizing and mitigating interconnected stressors that affect their health and QoL. 

In the national survey, our results showed that levels of infant well-being had a similar score compared to the original ColiQ [6], although the parents’ well-being score showed a lower impact of excessive crying on their QoL.

These results are in agreement with the literature that periodontitis plays an important role in the effect of oral health status on a person’s QoL [26,27,28]. Mothers who reported higher infant discomfort also showed less professional dental care prioritization, physical pain, and psychological and social disabilities. As pointed out in previous studies, excessive crying is one of the most stressful challenges for new parents, increasing parental stress, depression, and anxiety [11,12]. Consequently, higher perceived stress and/or depression are associated with poorer oral health through two main mechanisms [29]. While biological components imply a high allostatic load that influences baseline pathways for disease progression [30,31], the behavioral domain involves the tendency of individuals to engage in harmful patterns (use of alcohol, tobacco, improper diet, physical inactivity, and poor oral hygiene) that may lead to oral problems [32]. Once again is underlined how social and physical environmental factors contribute to oral health conditions [33].

Regarding possible risk factors for IC, our analysis revealed that younger infants had a slightly higher risk of being diagnosed with colic. This result is in agreement with the literature, indicating that IC reaches its highest intensity in the first 5/6 weeks of an infant’s life and progressively decreases until it disappears almost completely at approximately 3 months of age [34,35]. Parental-related factors, such as being unemployed and high educational levels, were associated with higher odds of IC. Conversely, prior investigations have found that a lower educational level is a predictor of excessive crying in infants [36,37,38]. It is worth highlighting that the majority of our participants (88.1%) had a university degree, therefore this result may be biased and should be carefully considered. On the other hand, unemployment is considered a strong indicator of elevated rates of psychological distress [39,40,41], and evidence has shown that psychological factors of parents may play an important role in parents’ IC perception [12,37,42]. In addition, excessive crying that characterizes IC also acts as a parental stressor [11,43]. Therefore, the established colic–parental psychological stress relationship appears to be bidirectional, although the mechanism is still poorly understood [44]. Thus, our study confirmed the multifactorial nature of IC, including infant-related and parent-related factors [34,44,45]. 

The successful validation on the ColiQ-PT, along with its straightforward applicability is relevant for the Portuguese scenario considering excessive crying in infants is reported to have a 40% prevalence in Portuguese 0-to-3-month infants [46]. Excessive crying pattern is also associated with lower levels of perceived maternal confidence [46]. This tool can aid parents in quantifying and articulating their concerns to healthcare professionals, as well as enabling them to actively assess and document their infants’ behavior and its impact on their own QoL. This can empower parents to play an active role in tracking their infants’ progress and becoming more involved in the therapeutic process. Therefore, this validation expands research and public health opportunities in Portugal, furthering the comprehensive understanding of the consequences of infant crying on parents’ QoL and facilitating the development of future behavioral health intervention strategies.

### Strengths and Limitations

The strengths of this study are worth discussing. First, the translation to Portuguese was performed by experts, following a rigorous linguistic validation guidance of clinical outcome assessment, and was approved by the entity that has the intellectual property of the original ColiQ. Second, the sample for the test–retest and validation phases was a consecutive pool of newly incoming women at the puerperium service of Garcia da Orta Hospital. Third, the national survey via a letter by the Lisbon Baby Lab using data from the National Registration Institute contributes to the generalizability of our results to the entire Portuguese population. Fourth, the relatively brief extension of the ColiQ-PT may contribute to favorable return and completion rates [47]. This feature may also enhance the interest and relevance of daily clinical practice. Also, in a world where the core of healthcare is increasingly focused on the patient, self-reported parameters, such as oral health status and OHRQoL, are considered pivotal to evaluate the consciousness and level of comfort concerning their oral health condition [48,49]. Self-reported questionnaires have proven to be a practical, useful and easy-to-use method [20,21,22]. It therefore appears to be an appropriate tool for monitoring the oral health status and OHRQoL of this sub-population of mothers, particularly in the first months after childbirth. Whether and how the ColiQ-PT can be implemented and its subsequent impact on public health is a matter to be analyzed. Its potential advantages, such as the possibility of being answered online, quickly and from the comfort of any digital device, make it a very interesting instrument to improve communication channels connecting health professionals and parents with colicky babies [6].

In what limitations concern, those results can only be interpreted as measures of surveillance as self-reported data never replace clinical diagnoses, whose accuracy and reliability are the gold-standard [50], particularly for periodontitis [51]. Furthermore, the methodology employing self-reported data collection may increase the likelihood of information bias. 

The ColiQ-PT used in this study serves as a tool for parents to monitor infants’ crying patterns rather than providing a clinical diagnosis of colic. This distinction is important, as the data collected from such questionnaires may not capture the full clinical picture of IC, potentially leading to the under- or over-estimation of its prevalence. Furthermore, the assessment of feeding practices, without utilizing a validated questionnaire, introduces another limitation. Our results diverged significantly from those published in the national and international literature [52], underscoring the need for a comprehensive national study using validated tools to ensure consistency and accuracy in future research.

## 5. Conclusions

Perceived infant discomfort seems to be linked to maternal deteriorated OHRQoL, particularly on the social disability and physical pain domains. This study underscores the necessity of prioritizing oral health in postpartum care. Additional research is required to investigate the underlying mechanisms of this association and to devise targeted interventions.

## Figures and Tables

**Table 1:** Participant sociodemographics and behaviors (*n* = 421).

Variables	Total
**Maternal age in years, mean (SD)**	32.9 (4.1)
**Education Level, *n* (%)**	
Elementary	3 (0.7)
Middle	47 (11.2)
Higher	371 (88.1)
**Marital status, *n* (%)**	
Married	357 (84.8)
Divorced	2 (0.5)
Single	62 (14.7)
**Occupation, *n* (%)**	
Employed	396 (94.1)
Unemployed	22 (5.2)
Student	3 (0.7)
**Smoking Habits, *n* (%)**	
Never	317 (75.3)
Active smoker	23 (5.5)
Former Smoker	81 (19.2)
**Brushing frequency (daily), *n* (%)**	
Less than once	13 (3.1)
Once	74 (17.6)
Twice or more	334 (79.3)
**Interproximal cleaning, *n* (%)**	
Yes, in all teeth	122 (29.0)
Yes, partially	118 (28.0)
No	181 (43.0)
**Infant feeding practice, *n* (%)**	
Breastfeeding	317 (75.3)
Mixed feeding	78 (18.5)
Formula	26 (6.2)
**Infant age (days), mean (SD)**	48.3 (21.8)
**Infant sex, *n* (%)**	
Girl	200 (47.5)
Boy	221 (52.5)
**ColiQ-PT Score, *n* (SD)**	
Infant	60.9 (18.0)
Parental	65.8 (18.6)
Total	63.3 (16.4)

**Table 2:** ColiQ-PT infant, parent and total score comparison according to infant sex, type of infant feeding practice, maternal oral-health-related quality of life (OHRQoL) affectance, and self-reported periodontal status (*n* = 421).

	ColiQ-PT					
Variables	Infant	*p*	Parent	*p*	Total	*p*
**Infant sex**						
Boy (*n* = 200)	60.9 (18.6)	0.984	64.1 (18.7)	0.052	62.5 (16.7)	0.139
Girl (*n* = 221)	60.9 (17.3)		67.6 (18.6)		64.2 (16.2)	
**Type of infant feeding practice**						
Breastfeeding (*n* = 317)	60.7 (17.1)	0.943 (#)	66.1 (18.7)	0.779 (*)	63.4 (16.1)	0.942 (#)
Mixed feeding (*n* = 78)	61.0 (19.9)		64.5 (18.0)		62.8 (17.3)	
Formula (*n* = 26)	61.9 (22.7)		65.5 (19.4)		63.7 (18.5)	
**OHRQoL affectance**						
Frequently affected (*n* = 108)	60.0 (18.7)	0.582	64.0 (19.1)	0.259	62.0 (17.2)	0.348
Not frequently affected (*n* = 313)	61.1 (17.8)		66.4 (18.4)		63.8 (16.2)	
**Self-reported periodontal status**						
Periodontitis (*n* = 82)	57.9 (18.8)	0.091	62.7 (18.9)	0.091	60.3 (17.0)	0.091
Healthy (*n* = 339)	61.6 (17.7)		66.5 (18.4)		64.1 (16.2)	

* values presented as mean (standard deviation). Mean score comparison by Student’s *t*-test and ANOVA (#).

**Table 3:** Oral Health Impact Profile-14 (OHIP-14) score comparison according to self-reported periodontal status (*n* = 421).

	Self-Reported Periodontal Status			Type of Infant Feeding Practice			
OHIP-14	Periodontitis (*n* = 82)	Healthy (*n* = 339)	*p* *	Breastfeeding (*n* = 317)	Mixed feeding (*n* = 78)	Formula (*n* = 26)	*p* #
Total	8.5 (9.2)	4.3 (6.1)	**<0.001**	5.2 (7.2)	5.3 (6.6)	3.9 (5.9)	0.645
Functional Limitation	0.6 (1.3)	0.2 (0.6)	**0.002**	0.3 (0.8)	0.3 (0.9)	0.1 (0.4)	0.591
Physical Pain	2.6 (2.2)	1.6 (1.8)	**<0.001**	1.8 (1.9)	1.9 (1.7)	1.7 (2.1)	0.796
Psychological Discomfort	1.7 (2.1)	0.8 (1.6)	**<0.001**	1.0 (1.7)	1.1 (1.9)	0.8 (1.9)	0.726
Physical Disability	1.1 (1.7)	0.7 (1.3)	**0.024**	0.8 (1.5)	0.6 (1.1)	0.7 (1.3)	0.528
Psychological Disability	1.2 (1.8)	0.6 (1.2)	**0.007**	0.7 (1.4)	0.8 (1.5)	0.4 (0.9)	0.385
Social Disability	0.5 (1.1)	0.2 (0.7)	**0.011**	0.2 (0.8)	0.2 (0.8)	0.2 (0.8)	0.842
Handicap	0.6 (1.3)	0.2 (0.8)	**0.021**	0.3 (0.9)	0.4 (0.9)	0.1 (0.4)	0.462
OHIP-14 Physical	4.9 (4.6)	2.5 (3.0)	**<0.001**	2.9 (3.5)	3.2 (3.6)	2.5 (3.8)	0.613
OHIP-14 Psychological	2.5 (3.3)	1.4 (2.5)	**0.006**	1.7 (2.8)	1.5 (2.2)	1.1 (2.1)	0.443
OHIP-14 Social	1.1 (2.3)	0.4 (1.3)	**0.011**	0.6 (1.6)	0.6 (1.6)	0.3 (1.2)	0.609

* Mean score comparison by Student’s *t*-test. # Mean score comparison by ANOVA. Significant (*p* < 0.05) differences denoted in bold.

**Table 4:** Correlation between ColiQ-PT infant, parent and total scores and maternal oral health self-reported outcomes (OHVS and OHIP-14).

		ColiQ-PT		
Variables		Infant	Parent	Total
OHVS	Total	−0.025 (0.605)	0.045 (0.352)	0.005 (0.912)
	Professional Dental Care	−0.053 (0.279)	−0.096* (0.050)	−0.079 (0.107)
	Appearance and Health	0.019 (0.693)	0.091 (0.062)	0.051 (0.298)
	Flossing	0.002 (0.970)	0.073 (0.132)	0.042 (0.386)
	Retaining Natural Teeth	−0.020 (0.688)	−0.029 (0.555)	−0.034 (0.488)
OHIP-14	Total	−0.036 (0.458)	−0.080 (0.101)	−0.064 (0.192)
	Functional Limitation	−0.051 (0.296)	−0.029 (0.547)	−0.045 (0.358)
	Physical Pain	−0.064 (0.191)	−0.101* (0.038)	−0.090 (0.064)
	Psychological Discomfort	−0.008 (0.877)	−0.047 (0.340)	−0.028 (0.563)
	Physical Disability	−0.027 (0.579)	−0.007 (0.879)	−0.017 (0.735)
	Psychological Disability	−0.049 (0.312)	−0.107* (0.029)	−0.089 (0.067)
	Social Disability	−0.106* (0.029)	−0.130* (0.008)	−0.130* (0.007)
	Handicap	−0.029 (0.549)	−0.079 (0.106)	−0.060 (0.223)
	OHIP-14 Physical	−0.048 (0.327)	−0.101* (0.038)	−0.082 (0.092)
	OHIP-14 Psychological	−0.019 (0.694)	−0.050 (0.302)	−0.036 (0.460)
	OHIP-14 Social	−0.044 (0.367)	−0.083 (0.088)	−0.072 (0.139)

* Correlation assessed by Spearman’s rank correlation coefficient (rho). Correspondent *p*-values also presented. Significant (*p* ≤ 0.05) correlations denoted in bold.

**Table 5:** Predictive multivariate logistic models (#) identifying the Odds-Ratio towards infant colic (lower ColiQ-PT values) (the outcome was dichotomized as high/low ColiQ-PT, based on median infant, parent and total score).

	*p*-Value	Odds-Ratio (OR)	OR (95% CI)
**Infant**			
Baby age (days)	**0.023**	0.987	0.977–0.999
Physical pain domain (OHIP-14)	**0.040**	1.149	1.006–1.311
**Parent**			
Education (higher) (*)	0.005	4.474	1.555–12.870
Occupation (unemployed) (**)	0.035	4.897	1.114–21.515
Physical pain domain (OHIP-14)	**0.017**	1.098	1.017–1.185
**Total**			
Social disability domain (OHIP-14)	0.031	1.515	1.039–2.208

CI—Confidence interval; OR reference: (*) Education (middle), (**) Occupation (employed).

## Data Availability

The data supporting the findings of this study are not publicly available due to ethical restrictions.

## Supplementary Materials

The following supporting information can be downloaded at: https://www.mdpi.com/article/10.3390/jcm13195931/s1; Table S1. Test−retest reliability using ICC for the ColiQ-PT, and descriptive statistics of the ColiQ-PT scores; Table S2. Model fit indices in the unifactorial model and configurational invariance by sex; Table S3. Correlation between ColiQ-PT subscale scores; Questionário de Cólica Infantil—ColIQ©.
