# Supplementary material for: Perceived Infant Discomfort Linked to Lower Maternal Oral Health Quality of Life: Results from a Cross-Sectional Study
Source: J Clin Med. 2024 Oct 5;13(19):5931. doi: 10.3390/jcm13195931 (PMC11477590; doi:10.3390/jcm13195931)
Supplement: Supplementary file 1 [file jcm-13-05931-s001.zip › jcm-3215422-supplementary.pdf]

Appendices

Supplementary Table S1. Test – retest reliability using ICC for the ColiQ-PT questionnaire, and descriptive statistics of the ColiQ-PT scores.

Variables	Correlation *	Cronbach a coefficient (95% CI)	ICC (95% CI)	Mean (SD)	Median (IQR)
**Symptom severity (Infant)**					
Sign 1	0.782	0.86 (0.65, 0.95)	0.74 (0.55, 0.86)	26.70 (34.21)	11.11 (44.44)
Sign 2	0.841	0.91 (0.84, 0.97)	0.84 (0.71, 0.91)	32.67 (29.30)	25.00 (50.00)
Sign 3	0.792	0.88 (0.73, 0.97)	0.79 (0.63, 0.88)	42.43 (19.52)	40.00 (30.00)
Sign 4	0.864	0.93 (0.79, 1.00)	0.87 (0.76, 0.93)	48.67 (29.67)	50.00 (0.00)
Sign 5	0.813	0.90 (0.79, 0.96)	0.82 (0.67, 0.90)	31.05 (18.45)	30.00 (27.50)
**Impact severity (Parent)**					
Impact 3	0.962	0.98 (0.94, 0.99)	0.96 (0.92, 0.98)	27.87 (22.28)	20.00 (30.00)
Impact 4	0.902	0.95 (0.77, 1.00)	0.90 (0.82, 0.95)	24.59 (30.72)	0.00 (40.00)
Impact 5a	0.833	0.91 (0.79, 0.98)	0.83 (0.70, 0.91)	44.27 (28.74)	50.00 (50.00)
Impact 5b	0.917	0.96 (0.9, 0.99)	0.92 (0.85, 0.96)	29.13 (32.44)	25.00 (50.00)
Impact 6	0.792	0.88 (0.66, 0.97)	0.80 (0.64, 0.89)	39.07 (26.56)	40.00 (40.00)
Infant Well-being score	0.916	0.96 (0.91, 0.98)	0.91 (0.84, 0.96)	63.70 (20.15)	67.00 (29.39)
Parent Well-being score	0.925	0.96 (0.90, 0.99)	0.93 (0.86, 0.96)	67.02 (20.80)	72.00 (29.00)
ColiQ-PT Total Score	0.935	0.97 (0.91, 0.99)	1.00 (-)	65.36 (18.83)	68.14 (25.68)

Abbreviations: CI - confidence interval; ICC - intraclass correlation coefficient; IQR - Interquartile range; SD - Standard deviation.

* Correlation was carried under Pearson correlation test.

Supplementary Table S2. Model fit indices in the unifactorial model and configurational invariance by sex.

Description	χ2	df	χ2/df	CFI	GFI	RMSEA (90% CI)	ΔCFI	Δχ2	Δdf
Unifactorial model	108.36	34	3.2	**0.946**	0.947	**0.076 (0.060-0.093)**	-	-	-

χ2, chi-square; df, degrees of freedom; CFI, comparative fit index; GFI, goodness-of-fit index; RMSEA, root mean square error of approximation; Δ, delta.

Supplementary Table S3. Correlation between ColiQ-PT subscale scores.

	S1	S2	S3	S4	S5	I3	I4	I5a	I5b	I6
**S1**	1.000	0.709*	0.164	0.479	0.552	0.382	0.055	0.309	-0.091	0.552
**S2**	-	1.000	0.733*	0.794*	0.164	-0.030	-0.418	-0.212	-0.430	0.018
**S3**	-	-	1.000	0.624	-0.273	-0.321	-0.576	-0.673*	-0.442	-0.345
**S4**	-	-	-	1.000	0.164	-0.479	-0.661*	-0.030	-0.806*	-0.212
**S5**	-	-	-	-	1.000	0.236	0.091	0.830*	-0.042	0.709*
**I3**	-	-	-	-	-	1.000	0.879*	0.212	0.648*	0.539
**I4**	-	-	-	-	-	-	1.000	0.273	0.733*	0.430
**I5a**	-	-	-	-	-	-	-	1.000	-0.006	0.576
**I5b**	-	-	-	-	-	-	-	-	1.000	0.527
**I6**	-	-	-	-	-	-	-	-	-	1.000

Values are the Spearman rank correlation coefficient (rho).

∗P < .05 mean statistical significance.

Questionário de Cólica Infantil – ColIQ©

Choro e desconforto / dor de estômago em bebés

Antes de preencher este questionário, por favor preencha a data de hoje:

/__/__/ /__/__/ /__/__/__/__/

Mês Dia Ano

Você é: ☐ A mãe do(a) bebé

☐ O pai do(a) bebé

Caros pais,

Este questionário foi elaborado com pais de bebés que choram e que sofrem de desconforto digestivo/dor de estômago. Ao preencher este questionário, ajudará o(a) profissional de saúde a entender-vos melhor, ao vosso bebé e a vocês. Isso permitirá que o(a) profissional de saúde forneça melhores cuidados médicos tanto para si como para o seu bebé.

Por favor, preencha este questionário sozinho e num lugar sossegado. Demore o tempo que precisar para responder.

Se não souber exatamente como responder a uma pergunta, escolha <u>a resposta que mais se aproxima à sua experiência ou à do seu bebé</u>. Não existem respostas certas ou erradas.

Obrigado por participar.

CHORO E DESCONFORTO DIGESTIVO / DOR DE ESTÔMAGO EM INFANTES

Para cada pergunta, marque a(s) caixa(s) que melhor descrevem sua experiência.

1. O meu bebé **chora**…	☐ Às vezes	☐ Muitas vezes
2. Quando o(a) meu(minha) bebé chora, **ele/ela chora durante**...	☐ Pouco tempo	☐ Muito tempo

3. Consegue estimar **quanto tempo é que o seu bebé chora**?

☐ Menos do que 1 hora por dia	☐ Entre 1 e 2 horas por dia	☐ Entre 2 e 3 horas por dia	☐ Mais do que 3 horas por dia

4. Das descrições a seguir, quais **descrevem melhor o choro do seu filho**?
(Mais de uma resposta é possível)

a. Grita	☐
b. Choro que parece um lamento aflitivo	☐
c. Choro prolongado	☐
d. Choro normal	☐

5. Das descrições a seguir, quais descrevem melhor como seu bebé se sente **quando chora e sofre de desconforto digestivo/dor de estômago**?
(Mais de uma resposta é possível)

a. O meu bebé tem a barriga inchada	☐
b. O meu bebé tem a barriga dura	☐
c. O meu bebé tem gases	☐
d. As pernas do meu bebé estão encolhidas sobre a barriga	☐
e. O rosto do meu bebé está vermelho	☐
f. O rosto do meu bebé está tenso	☐
g. O meu bebé tem os punhos cerrados	☐
h. O meu bebé está inquieto	☐
i. O meu bebé arqueia as costas	☐
j. O meu bebé contorce-se	☐

6. O meu bebé **parece estar com dor**	☐ Não de todo	☐ Um pouco	☐ Muita

CHORO E DESCONFORTO DIGESTIVO / DOR DE ESTÔMAGO EM INFANTES (continuação)

Nas perguntas abaixo, assinale a opção que melhor descreve o seu bebé (entre 0 = Nunca e 10 = Muitas vezes)

7. Quando o meu bebé chora, **eu não consigo acalmá-lo.**												
Nunca	☐0	☐1	☐2	☐3	☐4	☐5	☐6	☐7	☐8	☐9	☐10	Muitas vezes
8. O meu bebé está **inquieto quando está a dormir**.												
Nunca	☐0	☐1	☐2	☐3	☐4	☐5	☐6	☐7	☐8	☐9	☐10	Muitas vezes
9. Depois de dormir, **o meu bebé parece cansado**.												
Nunca	☐0	☐1	☐2	☐3	☐4	☐5	☐6	☐7	☐8	☐9	☐10	Muitas vezes
10. O meu **bebé chora por ter sido alimentado** (amamentação ou com biberão).												
Nunca	☐0	☐1	☐2	☐3	☐4	☐5	☐6	☐7	☐8	☐9	☐10	Muitas vezes

IMPACTO DO CHORO DO BEBÉ E DESCONFORTO DIGESTIVO/DOR DE ESTÔMAGO NA VIDA DOS PAIS

Por favor, assinale a opção das situações abaixo que melhor descrevem a sua experiência.

1. Quando o meu bebé chora, **para acalmá-lo**…	(Mais de uma resposta é possível)	
a. Eu deito-o de barriga para baixo com a barriga ao longo do meu antebraço	☐	
b. Eu dou-lhe um banho	☐	
c. Eu dou-lhe uma massagem	☐	
d. Eu abraço-o	☐	
e. Eu seguro-o pele-a-pele	☐	
f. Eu dou-lhe o peito ou o biberão	☐	
g. Eu faço outra coisa	☐	Explique :_______________
2. O que **tentou fazer** quando isso aconteceu?		
	(Mais de uma resposta é possível)	
a. Falei com o(s) profissional(is) de saúde	☐	
b. Eu dei-lhe um medicamento	☐	
c. Eu fiz outra coisa	☐	Explique :_______________
3. Quando meu bebé **chora e sofre de desconforto digestivo/dor de estômago**:		
	(Mais de uma resposta é possível)	
a. Acho a situação difícil	☐	
b. Eu sinto-me stressada(o)	☐	
c. Eu sinto-me sem esperança	☐	

d. Eu sinto-me culpada(o)	☐
e. Eu sinto-me inútil	☐
f. Eu sinto-me deprimida(o)	☐
g. Eu sinto-me desamparada(o)	☐
h. Eu choro	☐
i. Eu lido bem com isso	☐
j. Eu mantenho-me calma(o) sob controlo	☐

IMPACT OF BABY'S CRYING AND DIGESTIVE DISCOMFORT/STOMACH PAIN ON PARENTS' LIFE (continued)

4. **Devido** ao meu bebé **chorar e ter desconforto digestivo/dor de estômago, na relação enquanto casal** existe…	
	(Mais de uma resposta é possível)
a. Irritação	☐
b. Impaciência	☐
c. Tensão	☐
d. Discussões	☐
e. Sem alteração	☐
5. **Devido** ao meu bebé **chorar e ter desconforto digestivo/dor de estômago:**	
	(Mais de uma resposta é possível)
a. Durmo mal	☐
b. Não durmo o suficiente	☐
c. Sinto-me cansada(o)	☐
d. Tenho dores (nos meus braços, nas minhas costas, ...)	☐
e. Não tenho tempo suficiente para mim	☐
f. Não tenho tempo suficiente para cuidar das coisas em casa	☐
g. Não tenho tempo suficiente para a minha família ou amigos	☐
h. Não tenho tempo suficiente para sair e ver pessoas	☐
6. Diria que a **situação em que se encontra hoje é**:	
Fácil de lidar ☐0 ☐1 ☐2 ☐3 ☐4 ☐5 ☐6 ☐7 ☐8 ☐9 ☐10 Muito difícil de lidar	

Obrigado por ter preenchido este questionário.
